# GNSS Performance Modelling and Augmentation for Urban Air Mobility †

**DOI:** 10.3390/s19194209

**Published:** 2019-09-27

**Authors:** Suraj Bijjahalli, Roberto Sabatini, Alessandro Gardi

**Affiliations:** School of Engineering, Royal Melbourne Institute of Technology University, Melbourne, VIC 3083, Australia; suraj.bijjahalli@rmit.edu.au (S.B.); alessandro.gardi@rmit.edu.au (A.G.)

**Keywords:** Global Navigation Satellite System, error analysis, Urban Air Mobility, UAS Traffic Management

## Abstract

One of the primary challenges facing Urban Air Mobility (UAM) and the safe integration of Unmanned Aircraft Systems (UAS) in the urban airspace is the availability of robust, reliable navigation and Sense-and-Avoid (SAA) systems. Global Navigation Satellite Systems (GNSS) are typically the primary source of positioning for most air and ground vehicles and for a growing number of UAS applications; however, their performance is frequently inadequate in such challenging environments. This paper performs a comprehensive analysis of GNSS performance for UAS operations with a focus on failure modes in urban environments. Based on the analysis, a guidance strategy is developed which accounts for the influence of urban structures on GNSS performance. A simulation case study representative of UAS operations in urban environments is conducted to assess the validity of the proposed approach. Results show improved accuracy (approximately 25%) and availability when compared against a conventional minimum-distance guidance strategy.

## 1. Introduction

Several Location-Based Services (LBS) for personal navigation and Intelligent Transport Systems (ITS) are subject to the availability of robust positioning, navigation and timing measurements. The Global Navigation Satellite Systems (GNSS) provides a positioning solution that is free of drift as opposed to dead-reckoning systems such as the Inertial Navigation System (INS) [1,2]. However, due to a dependency on unobstructed line of sight between the mobile receiver antenna and the orbiting satellites, several advantages offered by GNSS are removed in dense urban environments. These environments are characterized by severe signal multipath and a high Dilution Of Precision due to unfavourable satellite geometry [3]. Rigorous navigation performance analysis is crucial for the safe operation of Unmanned Aircraft Systems (UAS). Roadmaps in different countries are aimed at the gradual introduction of UAS in previously restricted airspace including dense urban areas. Urban Air Mobility (UAM) is an emerging term encompassing not only UAS operations but also future on-demand urban air transportation (the so-called ‘air-taxi’). This emerging use-case is still in a conceptual stage at the time of writing. The efforts of most manufacturers in this domain are aimed at Distributed Electric Propulsion (DEP), Vertical Take-Off and Landing (VTOL) capability, and progressive automation of several safety-critical and mission-specific functions.

**Figure 1:**
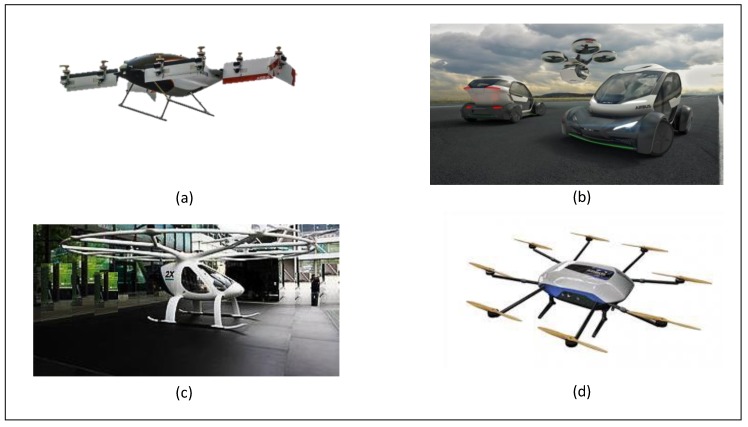
VTOL platform prototypes and concepts for Urban Air Mobility (**a**) Airbus Vahana (**b**) Airbus Pop.up (**c**) Volocopter (**d**) Airbus Skyways.

Initial offerings include (Figure 1) aerial logistics platforms such as the Skyways, and passenger aircraft such as the Airbus Vahana, Pop.up and the Volocopter. Initial feasibility studies [4] have been conducted that identify the key system requirements to be met for a pathway to market. Given the projected population of civilian UAS and UAM platforms [5], progressive introduction of autonomy is required for several functions such as monitoring trajectory conformance, envelope protection, and sensor performance to achieve feasibility from the perspective of pilot workload. Under this new paradigm, several locations in urban environments could emerge as favourable nodes for UAS and UAM platforms. This places capacity and safety requirements on low-level (below 500 feet) urban airspace. Conceptual airspace structures to accommodate these new users are currently being proposed under the UAS Traffic Management (UTM) framework, the European U-space and similar initiatives around the globe.

**Figure 2:**
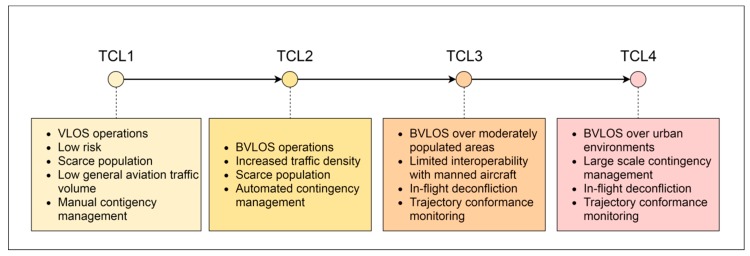
Phases of the UTM project.

Figure 2 illustrates the Technical Capability Levels (TCL) of the UTM undertaking. A successive progression from Visual Line Of Sight (VLOS) operations in scarcely populated areas to Beyond Visual Line Of Sight (BVLOS) operations in urban areas can be observed, highlighting the need for robust Communication, Navigation and Surveillance (CNS) systems.

Novel airspace structuring concepts are emerging in response to the drive to introduce UAM and UAS operations in urban environments. These include stacked layers [6], dynamic 4D tubes, designated zones [7], and urban air corridors. Urban Airspace access is likely to be based on aircraft capabilities including accurate, precise and reliable trajectory tracking and conformance, and robust Detect and Avoid (DAA) systems. These functions are highly dependent on the performance of the onboard navigation system. Navigation errors and uncertainties, in the first instance, lower the remote pilots’ situational awareness, and within a shared information network, ultimately propagate to cooperative surveillance systems, thereby lowering Separation Assurance and DAA capability. Therefore, persistent and high-performance navigation, and performance prediction are prerequisites for the implementation of a safe UAM framework. Most UAS navigation systems in the civilian market rely primarily on GNSS as the sole source of absolute positioning. Service coverage has also been expanded through the introduction of new constellations and multi-constellation receivers. In spite of the obvious benefits of GNSS-based positioning, several error sources and fault modes exist: signal propagation through the ionosphere and troposphere introduces ranging error that translates to receiver positioning error through the visible satellite geometry. In the urban environment, positioning availability and accuracy are adversely affected by receiver antenna obscuration and severe signal multipath.

This paper has two key objectives that are prerequisites for the implementation of a safe UAM framework. First, the model-based prediction of GNSS performance for operations in urban environments; Second, the development of a guidance-based augmentation strategy that allows the assessment of planned UAS trajectories in terms of navigation performance metrics. The scope of this paper is restricted to unintentional fault and error modes and an augmentation strategy that supports their mitigation. Beyond this scope, other important research initiatives that are necessary for fully realizing UAM operations include jamming and spoofing detection, cyber-security and Assisted-GNSS, which are not included in this work. This paper draws on work from [8,9,10], and adds to it comprehensive modelling of multipath/NLOS conditions based on ray-tracing which accounts for, in particular, geometrical and material properties of the urban environment, and of the airframe. The multipath modelling includes the effect of receiver tracking loop characteristics on overall positioning error. The NLOS/multipath models are used to characterize the expected GNSS error in urban canyon airspace. A path-planning strategy is developed which takes into account the expected error for the airspace and generates trajectories that avoid zones of degraded navigation performance. The remainder of this paper is structured as follows: Section 2 introduces the proposed system architecture followed by comprehensive modelling of GNSS performance in Section 3; Section 4 presents a simulation case study applying the developed methodology to a representative UAS operation in an urban environment, along with the relevant results.

### Related Work

A large body of knowledge exists on GNSS performance for aviation. A comprehensive performance analysis of GNSS performance for manned and unmanned aircraft including augmentation techniques was performed in [10]. Fault modes of GNSS and GNSS-INS integrated systems were identified and analysed in [11]. NLOS and multipath are accepted as being the dominant error sources in urban environments. As such, a number of methods have been developed to model signal reflections and their impact on positioning error. These methods can be either purely deterministic, which includes asymptotic methods such as ray-tracing [12,13], statistical [14], or hybrid methods [15]. A hybrid model based on ground vehicle measurement campaign data can be found in [16,17]. Regarding the simplification of the propagation environment, it has been found in [18] that simple canonical shapes representing building facades can be used to predict positioning error when wideband channel models are employed. GNSS coverage prediction models to adapt route-guidance are typically used in predictive-Receiver Autonomous Integrity Monitoring (p-RAIM). Planned routes can be assessed to determine whether GNSS failures can be detected based on satellite geometry projection [19]. A similar GNSS multipath prediction concept was used to drive path-planning in [20]. The risk associated with obstacles at low altitude flight was included as a cost-factor in an A* planning algorithm for a fixed wing aircraft in [21].

## 2. GNSS Augmentation Strategy

Navigation system performance has traditionally been quantified and evaluated through four metrics under the Performance Based Navigation (PBN) framework: accuracy, integrity, continuity and availability. An accuracy requirement places an upper limit on the maximum allowable difference between the estimated position and the true position. Accuracy requirements are specified as the maximum allowable 95% percentile of the position error. Integrity relates to the level of trust that can placed in the output of the navigation system, and is typically specified as a maximum allowable probability of the system providing hazardous and misleading information to the user. Integrity includes the ability of a system to provide timely and valid alerts to the user. Integrity requirements are described by the following metrics:

- Horizontal Alert Limit (HAL): Radius of a circle in the local horizontal plane (which is tangent to the ellipsoidal earth model) with its centre being at the true position, that describes the region that is required to contain the computed horizontal position with the required probability for a given navigation mode.
- Vertical Alert Limit (VAL): Half the length of a vertical segment, with its centre being at the true position, that describes the region that is required to contain the computed vertical position with the required probability for a given navigation mode.
- Time To Alert (TTA): The maximum allowable time measured from the onset of a positioning failure to the annunciation of the alert to the autopilot/mission-planner.
- Integrity risk: The probability of computing a position that is out of defined bounds without warning the autopilot/mission-planner within the TTA.

Continuity relates to the availability of the navigation system for a given phase of flight, and is typically specified as an allowable false alarm rate. Availability specifications are typically expressed as a minimum percentage of flight time for which the accuracy, integrity and continuity requirements must be met.

**Figure 3:**
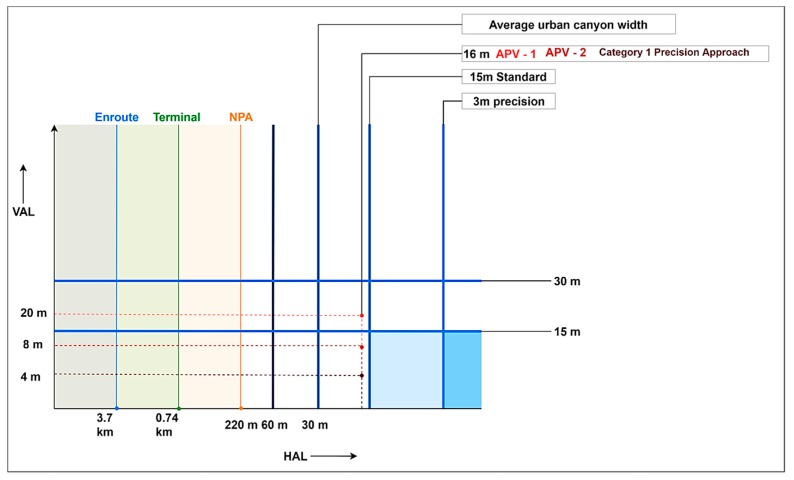
Alert limits for manned aircraft and preliminary PBN bounds for UAS.

Limiting values for each of these metrics are rigorously defined by ICAO for manned aircraft for different phases of flight. However, these standards cannot be directly applied to UAS since separation minima for unmanned aircraft do not currently exist. Aviation standards are in the process of evolving to accommodate UAS operations, and as such, performance requirements are not yet formally specified for this use-case. Even the standard approach RNP 0.3 (1823 ft/556 m) and minimum RNP 0.1 (607 ft/185 m) are too large for UAM and UAS platforms considering their intended applications. In a recent NASA study [22], a quantitative framework to adopt RNP to UAS applications based on operational risk was developed. An alternative source has been adopted in another NASA study in defining preliminary limits for navigation performance. The proposed limits are based on specifications derived by the Fédération Aéronautique Internationale (FAI) through years of practice and assessment of various model aircraft classes. Based on the FAI-derived safety boundaries, the preliminary standard horizontal PBN specification for UAS is set at 15 m, and the preliminary precision horizontal specification is set at 3 m. Nominal values of 30 m (standard) and 15 m (precision) were chosen for vertical PBN. These limit values are used in lieu of standardized alert limits in this paper, and form a volume bound within which positioning errors are to be contained. This is illustrated in Figure 3, comparing the assigned limits with the alert limits for manned aircraft. Integrity requirements in civil aviation are met through a fault detection system that is either implemented onboard the mobile platform itself, or external to the mobile platform. The most common strategy is to employ a three-layered approach in assuring integrity. Differential corrections and integrity flags are provided to the user by Satellite- and Ground Based Augmentation Systems (SBAS/GBAS) which essentially rely on external reference receivers to monitor positioning integrity and provide ‘do not use’ messages to the user when the estimated error exceeds the assigned limits [10].

**Figure 4:**
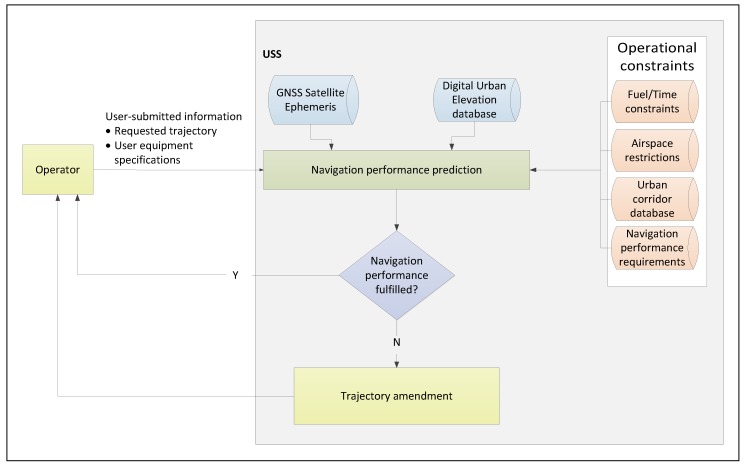
Proposed system architecture for GNSS-based UAM operations.

The third augmentation layer, Aircraft-Based Augmentation Systems (ABAS) implements monitoring functionality wholly within the user receiver itself, and is independent of the other two layers. ABAS is typically dependent on the availability of redundant sensor measurements to perform consistency checks and detect faults, although a model-based approach can also be used to detect or to predict loss of integrity [8,9]. ABAS benefits are twofold: first, the use of onboard sensors and/or models is free of dependency on external reference receivers as in the case of SBAS and GBAS; second: user-level monitoring is necessary to protect against localized error sources such as multipath, which cannot be mitigated by differential techniques like SBAS or GBAS. RAIM is one form of ABAS which relies on redundant GNSS measurements to detect and isolate faults. Owing to the capability to detect localised errors such as multipath (within a few wavelengths from the receiver antenna), ABAS assumes primary importance over SBAS and GBAS in the urban environment. Maintaining ABAS availability is therefore crucial to assure integrity in urban areas. Therefore, a UAS guidance strategy is presented that maximizes positioning accuracy accounting for propagation and user equipment –induced errors, and by extension system integrity and continuity. The approach is applicable in the strategic mission-planning phase to evaluate planned trajectories against sector-specific requirements. The overall proposed system architecture is depicted in Figure 4. UAS operators define their intended destinations or specify their preferred mission trajectory in the form of a request submitted to the UTM provider along with the equipped receiver specifications and characteristics.

The augmentation module outputs the expected navigation performance over the requested trajectory, and over the local area. In order to compute the expected navigation performance, the GNSS satellite orbits are projected over the intended mission duration. Urban elevation databases are also required to model signal interactions with buildings. The expected navigation performance is output to the trajectory generator in the form of discrete datapoints that vary over the operational area and over the duration of the mission. The datapoints convey expected accuracy and the number of visible satellites at a given location and time which relate to accuracy, integrity, continuity and availability requirements. The expected navigation performance is then included as an objective to be maximized in the trajectory generation module. The output trajectory is then the result of a trade-off between conventional objectives such as minimum fuel/ minimum time and navigation performance. The performance requirements for the given airspace sector are included as an operational constraint. This includes accuracy specifications, maximum allowable missed detection and false alarm rates, and percentage availability. The output of the trajectory generation module can either be an optimal trajectory that meets the assigned constraints, or a rejection of the trajectory altogether owing to an assessed non-compliance.

## 3. GNSS Performance Characterization

This section presents error models for GNSS, and in particular, analyses errors dominant in the urban environment viz. Non-Line of Sight (NLOS) and multipath errors. Secondly, the analysis is used to generate a guidance strategy that maximizes GNSS performance.

### 3.1. Error Modelling

The primary observable from a GNSS signal is what is termed the *pseudorange*, which is essentially the difference in time between when a signal is transmitted from the satellite, and the time at which it is received at the receiver antenna, multiplied by the speed of light. The reception of at least four such pseudoranges allows the receiver position to be determined, typically through a least-squares solution. The pseudorange essentially comprises the true range between the satellite and receiver, along with a number of errors and biases that contaminate the measurement and cause the estimated position to deviate from the true position. The model of the pseudorange observable from a single satellite is [10]:

(1) $$P=\rho +d_{\rho }+c\left(\mathrm{dt}-\mathrm{dT}\right)+d_{\mathrm{iono}}+d_{\mathrm{tropo}}+\varepsilon_{\mathrm{mp}}+\varepsilon_{\mathrm{noise}}$$

where c is the speed of light. $d_{\rho }$ jointly denotes the ephemeris and satellite clock errors. dt and dT are the satellite and receiver clock offsets, respectively. $d_{\mathrm{iono}}$ and $d_{\mathrm{tropo}}$ are the ionospheric and tropospheric delays, respectively. $\varepsilon_{\mathrm{mp}}$ is the code multipath error, which is a function of the relative geometrical configuration of the receiver and satellite antennas, and reflective surfaces in the local environment. $\varepsilon_{\mathrm{noise}}$ is the receiver thermal noise. ρ is the true geometrical satellite-receiver range at a given epoch given by:

(2) $$\rho =\sqrt{{\left(u^{p}-u_{r}\right)}^{2}+{\left(v^{p}-v_{r}\right)}^{2}+{\left(w^{p}-w_{r}\right)}^{2}}$$

where at any given epoch, $\left(u_{r},v_{r},w_{r}\right)$ are the Cartesian coordinates of the receiver and $\left(u^{p},v^{p},w^{p}\right)$ are the Cartesian coordinates of the satellite. Given a system of equations of the form of (1), the receiver coordinates (and GNSS time) can be derived from the simultaneous observation of four (or more) satellites. If more than four satellites are visible, a least-squares routine can be implemented to solve the over-determined set of equations. Each pseudorange error term in Equation 1 is described briefly.

#### 3.1.1. Ephemeris and Satellite Clock Errors

The satellite clock bias depends on the stability of the atomic clocks on the satellites, the control segment monitoring network and the latency of the corrections. Although these clocks are highly stable, the clock correction fields in the navigation data message are sized such that the deviation between SV time and GPS time may be as large as 1ms. The ground stations in the control segment determine and transmit clock correction parameters to the satellites for rebroadcast in the navigation message. These correction parameters are implemented by the receiver using the second-order polynomial [23]:

(3) $$dt^{p}=a_{f0}+a_{f1}\left(t-t_{oc}\right)+a_{f2}{\left(t-t_{oc}\right)}^{2}+\Delta t_{r}$$

where $a_{f0}$ is the clock bias; $a_{f1}$ is the clock drift; $a_{f2}$ is the frequency drift (aging); $t_{\mathrm{oc}}$ is the clock data reference time; t is the current time epoch; $\Delta t_{r}$ is the correction due to relativistic effects. This residual error is dependent on the Age Of Data (AOD), which is the time elapsed since the most recent control segment upload to a satellite. Residuals are the smallest following the upload, and then slowly degrade over time till the next upload.

The ephemeris for each satellite is estimated by the control segment and transmitted to the satellite along with other navigation data parameters for re-transmission to the user receiver. The ephemeris corrections are derived from a curve fit of the prediction of each satellites position at the time of upload. The contribution of ephemeris error to the overall pseudorange error is due to the residual error that remains in the satellite position after the curve-fit. This residual error is split into three components: Along Track (ATK), Across Track (XTK) and Radial (RAD). The maximum error occurs when the satellite lies in the plane of the horizon and the line-of-sight (LOS) user-satellite lies on the geometric plane containing ATK. In general, the error can be expressed as a function of the three components, in the form [10]:

(4) ERR=RADcosα+ATKsinα cosβ+XTKsinα sinβ

where α is the angle between the LOS user-satellite and the satellite vertical and β is the angle between the ATK direction and the plane containing the LOS and the satellite vertical. The ATK and XTK are more difficult for the control segment to observe through its monitors on the Earth’s surface, since these components do not project significantly onto the LOS(s) toward the Earth. The standard deviation of the pseudorange error model due to satellite clock and ephemeris inaccuracies is set to be equal to the satellite’s User Range Accuracy (URA), which is a one-sigma estimate of the satellite’s Signal-in-Space (SIS) User Range Error (URE).

#### 3.1.2. Atmospheric Errors

Atmospheric errors include both ionospheric and tropospheric delays. As the satellite signal passes through the ionosphere, there are two sources of delay: Firstly, propagation through a non-vacuum material delays the Pseudo Random Noise (PRN) codes while advancing the carrier phase. Secondly, signal refraction induces further delay. Error due to refraction can be neglected for most applications if the elevation of the satellite in question is 15° or more. The magnitude of the delay depends on the number of electrons that the signal encounters along its propagation path. Therefore, it is influenced both by the ionosphere characteristics, which vary depending on the time of day and the season, and the elevation angle of the satellite. An approximation of the ionospheric delay is given by [23]:

(5) $$\Delta \tau =40.31\frac{\mathrm{TEC}}{{\mathrm{cf}}^{2}}$$

where TEC is the total electron content integrated along the LOS to the satellite in units of electrons/m2, f is the frequency in Hz and c is the speed of light. TEC varies with time and depends on the location of the ionosphere ‘pierce-point’, or the intersection point of the LOS vector with the ionospheric layer. At the L1 frequency (1575.42 MHz), (5) can be written for a satellite that is not at the zenith:

(6) $$\Delta \tau =0.162\frac{F.{\mathrm{TEC}}_{v}}{c}$$

where ${\mathrm{TEC}}_{v}$ is the TEC value for a vertical column located at the pierce point, in units of 1016 electrons per m^2^ and is the obliquity factor. The active region of the ionosphere is typically represented as a thin shell at an elevation of 350 km, the obliquity can be approximated as a function of the satellite elevation angle in degrees (E):

(7) $$F=1+2.74\times 10^{-6}{\left(96-E\right)}^{3}$$

Depending on the receiver design, different models can be employed to compute the correction terms to be applied to the pseudorange before estimating position. L1 code-range receivers use a sinusoidal model of the ionosphere (the Klobuchar model), which accounts for the temporal variations of the ionospheric layers (low over night, rapidly increase after dawn, slow increase during the afternoon and rapid decrease after sunset). The sinusoidal parameters (amplitude and period) are transmitted in the navigation message:

(8) $$\mathrm{IDV}=\{\begin{array}{cc} \mathrm{DC}+\mathrm{Acos}\left[\frac{2\pi \left(t-\Phi \right)}{P}\right] & ,\;\mathrm{day}\\ \;\mathrm{DC} & ,\;\mathrm{night} \end{array}$$

where IDV (Ionospheric Vertical Delay) is expressed in nsec, DC is the constant night-day offset (5 nsec), A is the amplitude (whose value is between 10 and 100 nsec), Φ is the constant phase offset (14.00 hours), t is the local time, and P is the period. The two factors A and P are transmitted as coefficients of a cubic equation representing a model of the ionosphere with varying latitude. As the delay also depends on obliquity of the path, elevation is included as an additional factor in the equation [10]:

(9) $$\mathrm{TID}=\left[1+16{\left(0.53-E_{h}\right)}^{3}\right]\mathrm{IDV}$$

where TID is the Total Ionospheric Delay (nsec) and $E_{h}$ is the elevation angle of the satellites over the local horizon. As the ionosphere is dispersive at radio frequencies, the magnitude of the delay for two signals at different frequencies will be different. Dual frequency GNSS receivers (e.g., GPS P-code receivers) can therefore measure the difference between the time of reception of L1 (1575.42 MHz) and L2 (1227.60 MHz), and evaluate the delay associated with both of them. For L1 the delay is given by:

(10) $${\Delta \tau }_{L1}=\Delta T{\left[{\left(\frac{f_{L1}}{f_{L2}}\right)}^{2}-1\right]}^{-1}$$

where $\Delta T=40.31\frac{\mathrm{TEC}}{{\mathrm{cf}}^{2}}\;\left(\frac{1}{f_{L2}^{2}}-\frac{1}{f_{L1}^{2}}\right)$.

For single-frequency receivers, the Klobuchar model accounts for at least 50% of the root-mean-square raw code ranging error due to ionospheric delay. The residual code ranging error for GPS L1 C/A is given by:

(11) $$\sigma_{iono,L1}=F\tau_{v}$$

where F is the obliquity factor presented in (7), and $\tau_{v}$ is approximated from ${\mathrm{TEC}}_{v}$ to be:

(12) $$\tau_{v}=\{\begin{array}{c} 9m\;\mathrm{for}\;0\leq \left|\phi_{m}\right|\leq 20\\ 4.5m\;\mathrm{for}\;20\leq \left|\phi_{m}\right|\leq 55\\ 9m\;\mathrm{for}\;\left|\phi_{m}\right|>55 \end{array}$$

where $\phi_{m}$ is the geomagnetic latitude. For dual-frequency receivers, the iono-free pseudorange combinations remove the first-order ionospheric delay, but higher-order errors remain. However, since their magnitude is insignificant compared to other error sources, the residual ionospheric error for these types of receivers is assumed to be zero.

The troposphere is the lower part of the atmosphere extending up to approximately 50 km and its local propagations characteristics depend on several factors, including humidity, temperature and altitude. The tropospheric delay for a given slant range can be described as a product of the delay at the zenith and a mapping function, which models the elevation dependence of the propagation delay. In general, the total Slant Tropospheric Delay (STD) is given by the sum of a Slant Hydrostatic Delay (SHD) and a Slant Wet Delay (SWD), and both can be expressed by a relevant Zenith Tropospheric Delay (ZTD) and a Mapping Function (MF). The SHD (or “dry component”) accounts for about 90% of the total tropospheric delay and accurate estimations are normally available. On the other hand, the “wet component” (SWD) estimations are generally less accurate and there is a significant variability depending on the actual models implemented. The total STD is given by [10]:

(13) $$\mathrm{STD}=\mathrm{SHD}+\mathrm{SWD}\;\mathrm{STD}=\mathrm{ZHD}\times {\mathrm{MF}}_{H}+\mathrm{ZWD}\times {\mathrm{MF}}_{W}$$

where ZHD and ZWD are the zenith hydrostatic delay and zenith wet delay, respectively; (ZTD=ZHD+ZWD) are their corresponding MFs. A standardized and often employed from the RTCA for the residual error after troposphere correction is [10]:

(14) $$\sigma_{tropo}=\{\begin{array}{c} 0.12\times \frac{1.001}{\sqrt{0.002001+sin^{2}EL}}\;for\;El\geq 4{}^{\circ}\\ 0.12\times \frac{1.001}{\sqrt{0.002001+sin^{2}EL}}\left[1+0.015{\left(4^{{}^{\circ}}-El\right)}^{2}\right]\;for\;2{}^{\circ}\leq El\leq 4{}^{\circ} \end{array}$$

#### 3.1.3. Receiver Thermal Noise

The signal tracking module in any receiver comprises a Delay-Lock –Loop (DLL) which dynamically tracks the code delay of each incoming signal. Thermal noise at the receiver front-end perturbs this process and induces errors on the code pseudo-range measurement estimates, the magnitude of which is dependent on the employed DLL discriminator. The variance of the thermal noise ranging error is given by [24]:

(15) $$\sigma_{noise}^{2}=c^{2}\frac{B_{n}\left(1-0.5B_{n}T_{I}\right)\int_{-\frac{B_{fe}}{2}}^{\frac{B_{fe}}{2}}G_{s}\left(f\right)\sin^{2}\left(\pi f\Delta \right)df}{\frac{{\left(2\pi \right)}^{2}C}{N_{0}{\left(\int_{-\frac{B_{fe}}{2}}^{\frac{B_{fe}}{2}}fG_{s}\left(f\right)\sin\left(\pi f\Delta \right)df\right)}^{2}}}\cdot \left[1+\frac{\int_{-\frac{B_{fe}}{2}}^{\frac{B_{fe}}{2}}G_{s}\left(f\right)cos\left(\pi f\Delta \right)df}{T_{I}C/N_{0}{\left(\int_{-\frac{B_{fe}}{2}}^{\frac{B_{fe}}{2}}G_{s}\left(f\right)cos\left(\pi f\Delta \right)df\right)}^{2}}\right]$$

under the following assumptions:

- The Early Minus Late Power (EMLP) DLL discriminator is used.
- The receiver’s front-end filter is approximated by a rectangular band-pass filter centred at zero frequency and having two-sided bandwidth.
- The thermal noise is white with power spectral density

$B_{n}$ is the DLL bandwidth (Hz); $G_{s}\left(f\right)$ is the normalized signal power spectral density (/Hz); $C/N_{0}$ is the carrier to noise ratio; $T_{I}$ is the integration time; Δ is the early-late chip spacing; $B_{fe}$ is the bandwidth of the front-end.

#### 3.1.4. Multipath Errors

**Figure 5:**
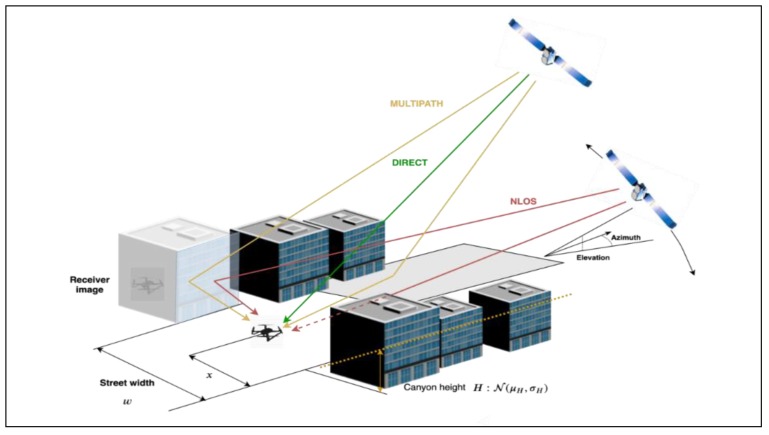
Satellite visibility and NLOS/multipath conditions in a typical urban environment.

In general, GNSS error components are assumed to be zero-mean and normally distributed. However, this assumption does not hold for multipath. A comprehensive investigation of terrestrial multipath based on several measurement campaigns was performed in [25,26]. The results are instructive from the perspective of urban environments. Echoes are classified either as near echoes caused by reflections in the foreground, with delays within 600 ns relative to the direct path, or as far echoes, with delays exceeding 600 ns. The multipath echoes distort the ideal correlation function of the code-tracking loop in the receiver, potentially leading to multipath pseudorange errors that are difficult to model. The magnitude of the multipath pseudorange errors depends upon not only the echo delays but also on the strength and phase of the echoes relative to the direct path, and the type of code-tracking loop employed in the receiver. The relative strength is characterized by the ratio of amplitudes of the multipath and direct rays – the Multipath to Direct Ratio (MDR), which in turn depends on the angle of incidence of the signal on the reflector, and the material properties of the reflector itself. The pseudorange error is directly proportional to the MDR value, with higher MDR values denoting a tendency to ‘overpower’ the direct ray. A distinction is made here between multipath and Non-Line of Sight (NLOS) instances. The ‘multipath’ instance refers to conditions in which the receiver has a direct line of sight to the satellite in addition to receiving one or more multipath echoes. As shown in Figure 5, in the NLOS condition, a direct line of sight does not exist.

If the NLOS ray is not significantly attenuated by interactions with objects in the local environment (reflection/diffraction), the receiver will be unable to reject the subsequently contaminated pseudorange from the navigation solution. NLOS conditions, therefore, can result in hazardously misleading information being provided to the vehicle guidance and control loops (when operating autonomously). The following signal reflection parameters directly impact pseudorange error:

- Relative delay: Additional distance (typically converted to time-delay) of the reflected signal relative to the direct signal;
- Relative amplitude: Amplitude of the reflected signal relative to the direct signal. This is typically in the range [0,1];
- Relative polarization: Reflection of the Right-Hand Circularly Polarized (RHCP) L1 wave from a perfect conductor results in a polarization reversal to Left-Hand Circularly Polarization (LHCP). RHCP receiver antennas can therefore partially mitigate multipath. However, since the urban environment has an unpredictable distribution of conductors and dielectrics, reflected signals will typically be a mix of right- and left-handed polarization, resulting in partial attenuation of the reflected signal.

The relative phase of the reflected signal impacts the fading characteristic of the overall received signal, but does not directly impact code-ranging error, and is therefore, not the primary focus of this paper. NLOS/multipath modelling approaches include numerical methods such as the traditional Method of Moments and parabolic methods, asymptotic methods such as ray-tracing, and hybrid stochastic-deterministic methods [17]. Driven by the need for accurate and precise positioning in urban road transport, a number of NLOS modelling methods based on ray-tracing and urban models have been published over the last two decades [13,27,28,29]. A more comprehensive survey of ray-tracing methods can be found in [30]. The approach in this paper is to model overall NLOS/multipath error as a function of three components: ground reflection, building façade reflection and UAV airframe reflection. Each of these components are addressed in turn.

#### 3.1.5. Ground Reflection

**Figure 6:**
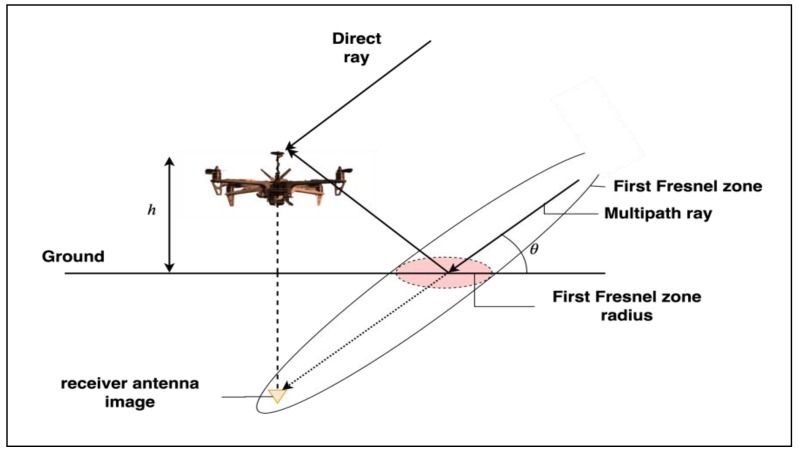
Ground reflection.

The first-order specular ground reflection model is illustrated in Figure 6. Assuming a locally flat ground plane, the so-called image theory from fundamental electromagnetics can be employed to determine the relative delay [31].

The delay in this case is the distance δ from the point of reflection to the image of the receiver antenna about the ground plane given by:

(16) δ=2hsinθ

where h is the height of the antenna and θ is the elevation of the satellite with respect to the ground plane. Significant specular reflection will occur only for reflectors with sizes comparable to the first Fresnel zone radius. The first Fresnel zone radius r is approximated by:

(17) $$r\approx \;\sqrt{\lambda d_{r}}$$

for cases where the distance between the transmitter and reflector is much larger than the distance from the reflector to the receiver ($d_{r}$). λ is the wavelength of the incident ray (≈ 19 cm for L1 in this case). A critical time delay $\tau_{crit}$ can be derived for different types of receivers (different in terms of correlator spacing). Multipath delays exceeding $\tau_{crit}$ will be rejected by the receiver. Standard receivers with a spacing of one Pseudo-Random Noise (PRN) bit between early and late correlators have a value of $\tau_{crit}\approx 1500$ nanoseconds. This corresponds to a distance of $\tau_{crit}\times c=$ 450 m. This value reduces to 1000 nanoseconds (300 m) for more advanced narrow correlator receivers with spacings of 0.1 PRN bit. The relative amplitude is a function of the reflection coefficients of the reflector material, which are in turn, dependent on the permittivity of the material and the angle of incidence of the incoming ray. The Fresnel reflection coefficient is resolved into perpendicular and parallel components as given by Kraus and Fleisch [31] for dielectrics:

(18) $$r_{⊥}=\frac{\cos\theta_{i}-\sqrt{\varepsilon_{r}^{*}-sin^{2}\theta_{i}}}{\cos\theta_{i}+\sqrt{\varepsilon_{r}^{*}-sin^{2}\theta_{i}}}$$

(19) $$r_{∥}=\frac{\varepsilon_{r}^{*}\cos\theta_{i}-\sqrt{\varepsilon_{r}^{*}-sin^{2}\theta_{i}}}{\varepsilon_{r}^{*}\cos\theta_{i}+\sqrt{\varepsilon_{r}^{*}-sin^{2}\theta_{i}}}$$

where $\varepsilon_{r}^{*}$ = $\varepsilon_{r}-j\frac{\sigma }{\omega \varepsilon_{0}}$; $\theta_{i}$ is the angle of incidence (relative to the surface normal); $\varepsilon_{r}$ is the relative permittivity of the lossy media; $\varepsilon_{0}$ is the absolute permittivity of free space; σ is the conductivity of the media. The reflectance is then:

(20) $$R=\frac{\left({\left|r_{⊥}\right|}^{2}+{\left|r_{∥}\right|}^{2}\right)}{2}$$

**Figure 7:**
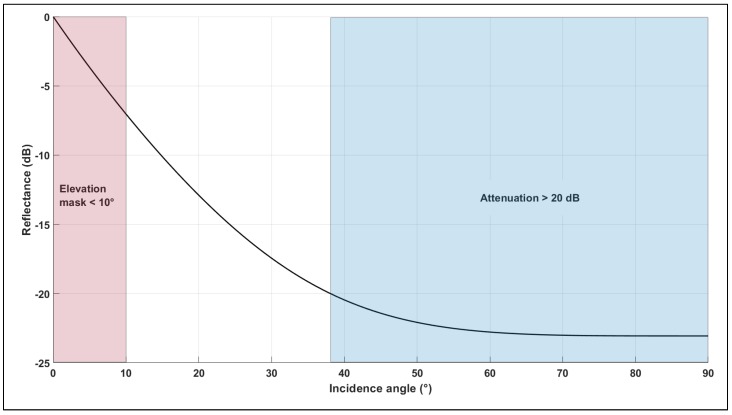
Ground reflectance region.

The following parameters ($\varepsilon_{r}=2.0,\;\sigma =0.12$) corresponding to asphalt were chosen to model the ground reflectance and are plotted in Figure 7 as a function of incident angle. A satellite elevation mask of 10° is assumed.

It is also assumed that signals attenuated greater than 20 dB cannot be acquired by the receiver. This corresponds to a multipath region of 10°~38°. Assuming an operational airspace sector from 30 m AGL to 150 m AGL, this corresponds to echo delays in the range 34~615 nsec.

#### 3.1.6. Building Facade Reflection

For the case of reflection from building facades, the receiver-image theory used in modelling the ground reflection is applied here as well. The employed ray-tracer is used to model multipath and NLOS conditions and quantify the corresponding delays and amplitudes. In order to verify if a multipath/ NLOS condition exists for a given satellite, reflector and receiver configuration, and to compute the corresponding reflectance and path-length difference, the following steps are applied:

- Compute the position of image of the receiver antenna in the reflector;
- Compute the vector from the satellite to the receiver image;
- Inspect whether the vector intersects the reflector. If such an intersection exists, a reflected signal exists in addition to the direct-path signal;
- If a direct ray exists between the receiver and satellite, the reflected ray is a multipath ray; If there is no direct line of sight, the reflected ray is an NLOS ray.

A similar approach has been employed in [32,33]. The building facades are discretised into triangular elements, and the Moller-Trumbore [34] algorithm commonly used in computer graphics applications is used to check for the intersection of rays with triangular elements. This type of ray-tracing model has previously been experimentally verified in [35]. More generally, these asymptotic methods are valid when the size of the obstacle is larger than the wavelength of the impinging wave [18]. A priori knowledge of the urban environment is required, and can be obtained from aerial views using stereo-photogrammetry, from digital cadastre data or by digitising analogue maps (as is the case for most radio channel modelling applications). The attainable accuracy of urban databases is typically on the order of 0.5 m (1σ) for the horizontal dimension, and is usually larger for vertical dimensions. It is also a common practice to prune unnecessary geometrical features from the database and to simplify the urban geometry into canonical shapes to minimize required computational resources. Accurately assigning electromagnetic parameters for each individual reflector surface is impractical and the commonly employed solution is to adopt effective electromagnetic parameters, i.e., parameters representative of the overall behaviour of the channel. The following parameters ($\varepsilon_{r}=5,\;\sigma =10^{-2}$) are usually employed for typical building walls in European cities [36]. A more conservative approach is to assume reflection from a metal surface in all instances as these are more common in urban environments (bridges and metal-clad buildings) and represent the worst-case scenario. This assumption means that virtually no attenuation occurs at the signal-reflector interface.

#### 3.1.7. Airframe Reflection

Signal reflections from the UAV frame is specific to each aircraft. An exhaustive approach is to apply a numerical technique such as the Method of Moments to a given airframe. Alternatively, in [15], a Physical Optics (PO) – based ray-tracer was applied to develop a model of fuselage reflection for large fixed-wing aircraft.

**Figure 8:**
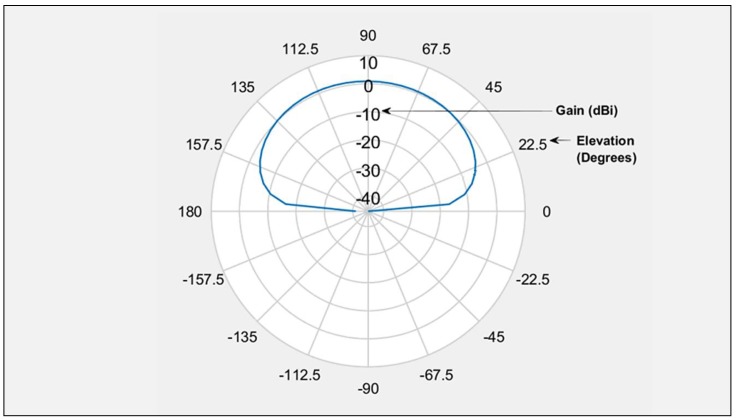
Microstrip patch antenna radiation pattern.

**Figure 9:**
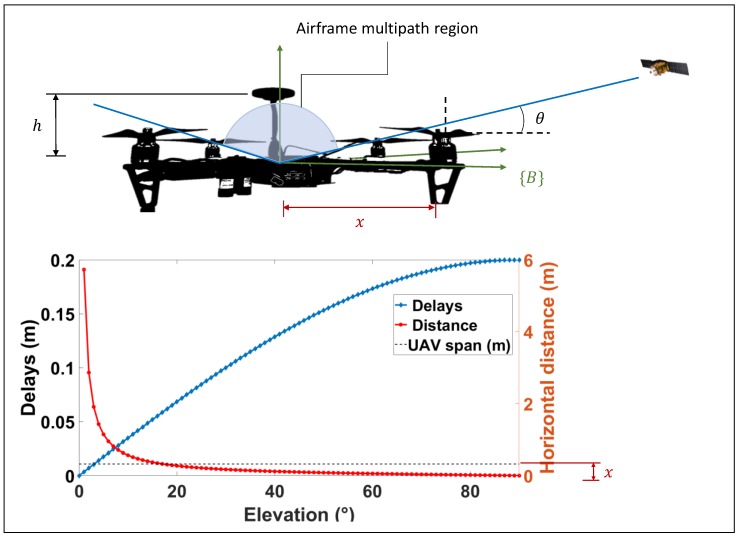
Multipath region for a small UAV (owing to reflections from the aircraft body).

The most obvious differences between the large-fixed wing aircraft scenario and present-day rotary wing aircraft lie in their physical dimensions, antenna placement configuration and material properties. Receiver antennas are typically sited 5–10 cm above the airframe, and are installed with a small ground plane to dampen the radiation pattern back lobe. This aids the minimization of the antenna gain for signals arriving from low elevation angles (such as signals reflected from the airframe). This is illustrated in Figure 8 for a microstrip patch antenna modelled with a dielectric substrate and small groundplane, as commonly installed on low-cost GNSS receivers. The figure shows the elevation gain pattern of the antenna for an azimuthal slice. It can be safely assumed that only the upper portion of the airframe contributes to the airframe multipath channel through a combination of reflection and scattering. Figure 9 depicts a reference platform that is broadly representative of most small to medium-sized UAV configurations on the market.

The largest delay will be from a low elevation satellite (relative to the UAV body frame {B}) and can be derived through the same trigonometric relationships used to model ground reflection delay. Knowledge of the maximum dimension of a given UAV allows an approximation of the range of possible echo delays from the body of the aircraft. A reference platform is adopted as shown in Figure 9 for the analysis.

The height of the antenna above the mounting bed and the half-span (center to motor distance) is known (h and x, respectively). The specular airframe reflection region (angular) is computed to be

(21) $$\theta \;\in \left[\tan^{-1}\frac{h}{x},\;90{}^{\circ}\right]$$

where θ is the elevation of the satellite in the UAV body frame {B}

The corresponding reflection delays are bounded by:

(22) δ ∈[2hsinθ,2h]

which simplifies to:

(23) $$\delta \;\in \left[\frac{h}{\sqrt{h^{2}+x^{2}}},2h\right]$$

For example, assuming a span of 650 mm measured from propeller to propeller (most commercial small to medium-sized UAVs range in size from 450-650 mm), it can be seen from Figure 9 that specular reflections are restricted to satellites at elevations (in the UAV body frame) from 17° and above, leading to delays in the range of 19.49 ns – 66.66 ns. This corresponds to the short multipath region as designated by [26]. Nominal values of ($\varepsilon_{r}=3.5,\;\sigma =2\times 10^{-12}$) corresponding to material parameters for polyamide thermoplastics commonly used in UAV airframes are assumed.

#### 3.1.8. Receiver Model

In addition to geometrical effects, pseudorange error also has a dependency on the receiver architecture and the specific type of discriminator function used to extract a pseudorange measurement from a satellite. The primary focus of this paper is the effect of multipath on the code-phase. Therefore, the receiver model implementation is limited to the Delay Lock Loop (DLL) aspects viz the employed discriminator function. In order to estimate the pseudorange error, the estimated power delay profile must be fed through the In-phase (I) and Quadrature-phase (Q) arms in order to form a discriminator function as demonstrated in [37]. In order to simplify the analysis in this paper, a weighted average is computed over all the echoes for a given receiver-satellite-reflector configuration at a given altitude in the urban canyon. This yields a single characteristic multipath ray. This allows us to employ a simplified single-ray multipath error envelope which maps the multipath delay and relative amplitude to the pseudorange error. The error is dependent on the two geometry-dependent parameters discussed so far, i.e., the multipath delay and the relative amplitude, and one receiver-dependent parameter, viz. the correlator spacing (specified in units of chips). Multipath error envelopes for Binary Phase Shift Keying (BPSK) (1) modulation for GPS are estimated by the following inequalities [38]:

(24) $$\tau =\frac{\alpha \delta }{\alpha +1};\;\mathrm{for}\;0\leq \;\delta \leq \frac{d}{2}\left(\alpha +1\right)\;\tau =\frac{\alpha \delta }{2};\;\mathrm{for}\;\frac{d}{2}\left(\alpha +1\right)\leq \;\delta \leq \left(1+\frac{d}{2}\right)\left(\alpha -1\right)\;\tau =\frac{\left(d-\frac{\delta }{2}-1\right)\alpha }{\alpha -2};\;\mathrm{for}\;\left[\left(1+\frac{d}{2}\right)\right]\left(\alpha -1\right)\leq \;\delta \leq 1+\frac{d}{2}$$

Standard correlator spacings of one chip (d=1 chip) have the largest potential pseudorange error. However, most modern receivers employ narrow correlators, where the spacing is 0.1 chip. Equation 16 assumes infinite bandwidth of the autocorrelation function. Modeling finite bandwidth would be more accurate in terms of the estimated pseudorange error. However, peak error is reduced when accounting for bandwidth in the model and therefore the infinite bandwidth assumption yields more conservative estimates [38]. The receiver model is used to set an upper bound on the pseudorange error based on the conservative estimates of delays and echo strengths for the ground and airframe reflections. For reflections from building faces, the error estimation is based on the more severe NLOS condition.

An urban canyon test domain of (L) 100m × (W) 50m is defined to model the described electromagnetic interactions with building facades. The following parameters are tunable in the implemented urban canyon model and are set to:

- Street width: 30m (common in the U.S, Europe and Australia)
- Number of buildings: 8
- Building widths: 50m
- Building spacing: 5m
- Building height: ($\mu_{h}:$ 65m $,\;\sigma_{h}:$ 5m)

A grid of receiver points is defined over the model domain with a horizontal resolution of 1m and vertical resolution of 5m up to a ceiling of 10m above the maximum building height. Satellite elevation is varied in increments of 10 ° from a masking angle of 10° to 90°. The previously described steps to check for NLOS/multipath conditions are applied for each angular increment.

**Figure 10:**
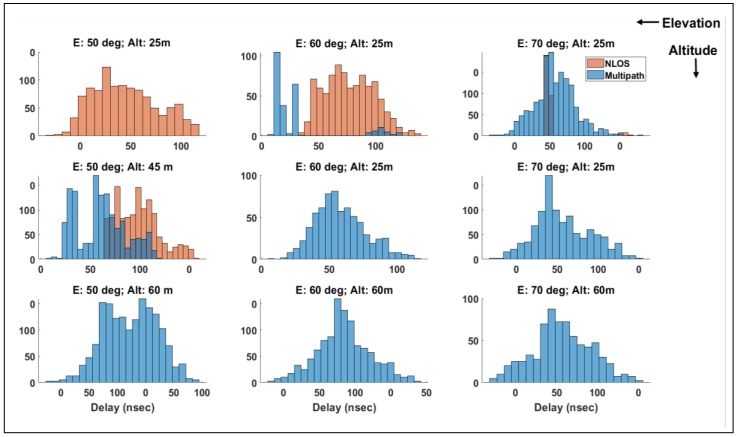
Histogram of NLOS and multipath delays for varying satellite elevations and altitude AGL in an urban canyon.

Figure 10 is a plot of histograms of delays for three altitudes and three satellite elevation angles. As expected, the likelihood of tracking an NLOS signal is strongly dependent on the altitude and satellite elevation. In general, NLOS conditions were met more frequently at low operating altitudes and low elevation angles. The overall error variance due to signal reflection is described by:

(25) $$\sigma_{r}^{2}=\sigma_{g}^{2}+\sigma_{b}^{2}+\sigma_{NLOS/multipath}^{2}$$

where $\sigma_{g}^{2}$, $\sigma_{b}^{2}$, $\sigma_{NLOS/multipath}^{2}$ are the contributions from the ground, UAV body and buildings, respectively. Each of these components is modelled as functions of altitude h and elevation θ, and a conservative bound on each of the three components is set by worst-case delay and reflectance assumptions. The User Equivalent Range Error (UERE) or the statistical aggregate of the errors described so far is then formulated as:

(26) $$\sigma_{\mathrm{UERE}}\left(h,\theta \right)=\sqrt{\sigma_{c/e}^{2}+\sigma_{\mathrm{iono}}^{2}+\sigma_{\mathrm{tropo}}^{2}+\sigma_{\mathrm{noise}}^{2}+\sigma_{r}^{2}}$$

It can be observed that the UERE captures both the errors allocated to the space and control segments viz the User Range Error (URE), and the errors allocated to the user equipment viz the User Equipment Error (UEE). The UERE at any given epoch is dependent on the time lapsed or the Age of Data (AOD) since the last upload of the satellite clock and ephemeris corrections from the control segment to the space segment. UERE, therefore, is not temporally static and grows in a predictable manner from the navigation message upload to the maximum AOD under normal operating conditions (Approximately 24 hours). GNSS UERE budgets therefore tend to vary widely depending on assumptions made regarding receiver design and the operating conditions. Apart from the UERE, the receiver position uncertainty also depends on the relative geometry of the satellites and the user receiver. This is characterized by the Dilution of Precision (DOP) factors, which are presented here without the full proof. The reader is directed to [10] for a detailed derivation of the DOP factors. The covariance matrix $C_{\bar{x}}$ of the position error represents the uncertainty in the position and is related to the UERE by the expression [10]:

(27) $$C_{\bar{x}}=PDOP.\sigma_{UERE}{}^{2}$$

(28) $$PDOP=\sqrt{D_{11}+D_{22}+D_{33}}$$

The parameters $D_{\mathrm{ii}}$ on the right-hand side of Equation 14 are the diagonal elements of the geometry matrix:

(29) $$D={\left(H^{T}H\right)}^{-1}$$

where H is the observation matrix from comprising the unit LOS vectors from the receiver to the visible satellites. The DOP parameters are important in quantifying the effect of satellite geometry on the accuracy of the solution. These parameters are computed in-flight and can also be predicted for a specified flight profile, given knowledge of satellite orbits and terrain models. DOP prediction services are now being offered by select USS as part of pre-flight planning.

Equation 27 essentially represents positioning uncertainty as a function of the pseudorange errors discussed so far, the number of visible satellites and the geometry of the visible satellites.

The predicted GNSS error over the test domain is used to define a cost-map that is applied to the guidance problem. Several options are applicable for this use-case including the well-known A* algorithm [39]. This is an extension of the Djikstras algorithm for finding the least-cost path between designated start and destination grid cells. An a priori map of the operational environment is assumed, which is parameterized as a 2D grid. The occupancy of each cell is a Boolean value, with {0} corresponding to free space, and [40] corresponding to occupied.

The cost of traversing a cell n is then:

(30) f(n)=g(n)+h(n)

where g(n) is the running cost incurred upto cell n i.e., the number of nodes traversed upto that point; h(n) is an estimate of the remaining c ost to travel from cell n to the specified goal cell.

h(n) is a user-specified heuristic. In this paper, the Euclidean distance metric is employed:

(31) $$h\left(n\right)=\sqrt{{\left[x\left(n\right)-x\left(goal\right)\right]}^{2}+{\left[y\left(n\right)-y\left(goal\right)\right]}^{2}}$$

f(n) is augmented with an added cost which is associated with the predicted navigation error $C_{\bar{x}}\;\left(n\right)$:

(32) $$\bar{f\left(n\right)}=\;f\left(n\right)\left[1+\tilde{C_{\bar{x}}}\;\left(n\right)\right]$$

where $\tilde{C_{\bar{x}}}\;\left(n\right)$ is the navigation error of cell n normalized against the maximum navigation error at a given operational altitude.

## 4. Simulation Case Study

A UAS operation in an urban environment is chosen as a case study. The models presented in Section 3 are used to develop a path-planning cost-map that accounts for navigation performance and distance covered. Multirotor aircraft have rapidly grown in popularity for a number of commercial and research applications. Among the different possible multirotor configurations, quadrotors are particularly widespread and support a number of indoor and outdoor applications. The conducted case study simulates a low Size, Weight, Power and Cost (SWaP-C) quadrotor in a representative mission in an urban environment. However, the overall approach can be generalized to other UAS as well. Quadcopter dynamics are well documented in the literature and are modelled as in [41]. The simulations were performed in a MATLAB™ environment to investigate GNSS performance and the developed path-planning strategy in a representative urban environment. The simulation scenario was developed using CAD models of urban environments, which were converted to a STereoLithographic (STL) format to import into MATLAB™, to simulate realistic geometry. Realistic satellite orbits were simulated using a YUMA almanac.

RMS accuracy was computed over the test domain for the intended flight profile. Two scenarios were studied: (1) A single-frequency receiver scenario wherein ionospheric error contributed significantly to the overall UERE owing to the relative inaccuracy of the employed ionospheric model; (2) A dual-frequency receiver scenario wherein ionospheric error is practically eliminated. In this scenario, multipath contributions are the limiting source of error. In each scenario, a conventional A* algorithm is run to generate a baseline trajectory, and is compared against a navigation cost-augmented A* algorithm. The comparison is on the basis of two parameters: Average accuracy over the trajectory, and percentage availability. A representative urban environment is simulated along with a satellite constellation. An *a priori* known binary occupancy grid map is assumed for the navigation area i.e., the environment is represented in discrete voxel form with labels corresponding to either occupied (Boolean-1) or free-space (Boolean-0). This provides a straightforward means of spatially mapping GNSS parameters over the urban airspace. An urban canyon was simulated since most dense urban environments are characterized by this type of scenario. The error models presented in Section 3 were used to generate a spatial and temporal distribution of range error over the simulated domain.

**Figure 11:**
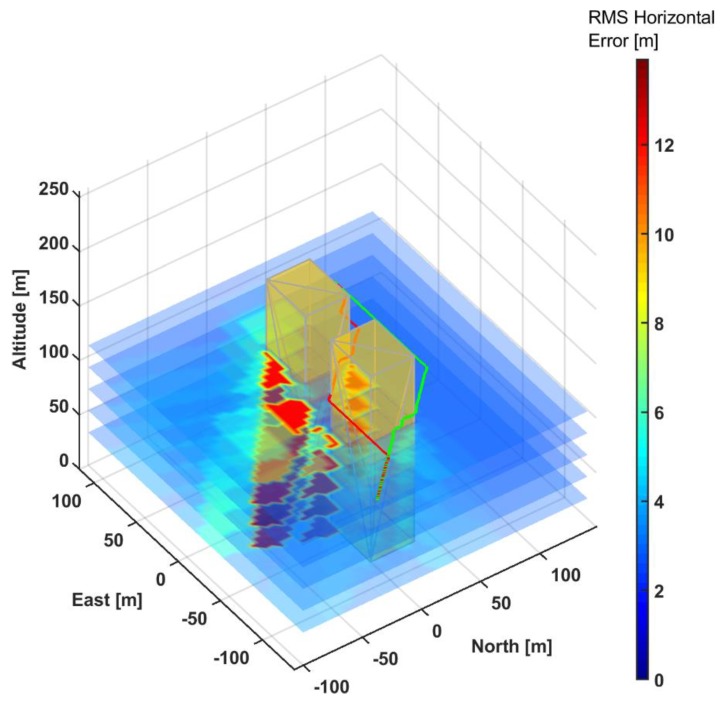
HRMS accuracy field along with baseline A* and Nav-A* trajectories.

In particular, the NLOS/multipath error was focused upon owing to its high variability with satellite elevation and azimuth, and receiver altitude. In all the simulated operations, aircraft path-planning was performed using the A* algorithm, which is widely employed in aerial robotics applications and is guaranteed to converge to the least-cost or shortest path given start-end points and obstacle locations. Figure 11 illustrates the test-domain in the single-frequency receiver scenario. The expected RMS horizontal accuracy field as evaluated at each grid point is shown overlaid over the domain.

**Figure 12:**
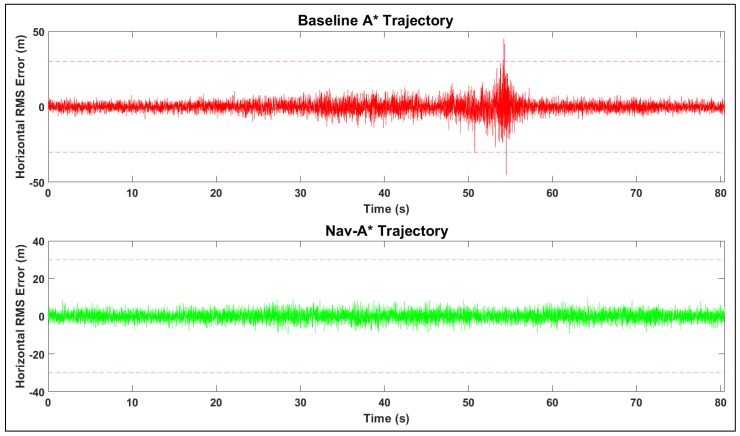
HRMS error time-series (Dual-frequency).

As expected, accuracy is degraded in the low-altitude shadowed regions of the test domain owing to poor geometry and larger multipath contributions. Figure 11 also shows the baseline A* algorithm along with the navigation cost-augmented path (Nav-A*). The simulation results are summarized in Table 1. The Nav-A* trajectory horizontal error is approximately 25% lower than the baseline A* trajectory. The average accuracy improves to approximately 2.8 m for the dual frequency scenario owing to the near-elimination of ionospheric error. The trajectory generated by the Nav-A* algorithm clearly avoids the dropout zones incurred by the baseline A* algorithm. The time-series of the RMS Horizontal RMS (HRMS) error for both trajectories is displayed in Figure 12.

**Figure 13:**
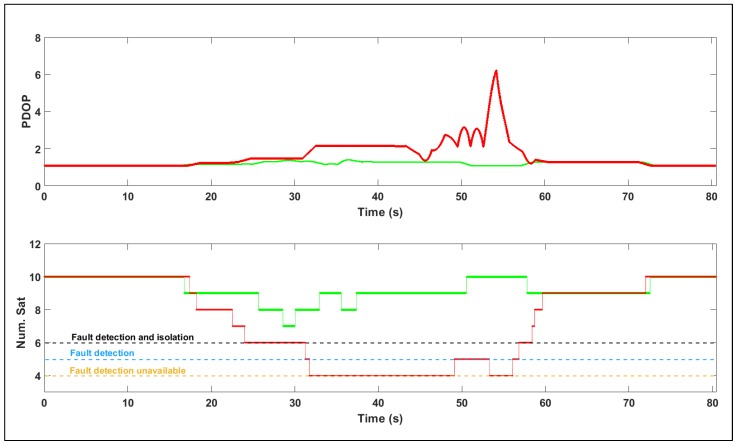
PDOP and Number of visible satellites for A* and Nav-A* paths.

Minimum performance requirements have not been standardised for UAM navigation systems at the time of writing. Therefore, a limiting threshold value approximately equal to one-third of an average urban street width (10 m) is set to compute the system availability, which lies between the standard and precision PBN boundaries presented in Section 2. Figure 13 plots the PDOP and number of visible satellites for both trajectories. The number of redundant visible satellites dictates the fault detection and isolation capability of onboard systems such as RAIM. As described in [3], a minimum of six satellites is required for RAIM-based fault detection and isolation. Only fault detection is possible with five visible satellites, and integrity monitoring is unavailable with only four visible satellites. The availability is computed as the percentage of time over the flight for which the accuracy, integrity and continuity requirements are jointly satisfied. This inclusion of navigation parameters in the path-planning cost map has clearly resulted in an improvement in overall system availability over the trajectory. However, this improvement is marginal in the single-frequency scenario since the ionospheric error dominates in this scenario, and is considered uniform over the relatively small test-domain. The navigation performance-augmented path incurs a penalty in terms of distance, but minimizes accuracy violations as compared to the conventional A* algorithm.

## 5. Conclusions

GNSS performance degradation in urban environments is a critical challenge to overcome to support safe urban air mobility. A focused study on GNSS performance for UAS navigation in urban areas was proposed and numerically assessed. The analysis supported the development of a framework which allows prediction of performance and a corresponding trajectory generation strategy. The key GNSS error sources and fault modes affecting navigation performance in urban environments were presented, along with metrics for predicting navigation performance over short UAS missions. In particular, NLOS/multipath characteristics in an urban canyon scenario were modelled, including the impact of building facade, ground and airframe reflections. A guidance strategy accounting for the predicted GNSS performance in urban airspace was developed. The presented methodology allows the assessment of trade-offs between distance and navigation performance in dense urban environments. User-requested routes can be assessed by the UTM operator against the required navigation performance for a given airspace sector and either accepted/rejected/amended. The methodology utilizes 3D models of the navigation environment, GNSS almanacs and models characterizing receiver characteristics to predict positioning error over a specified mission timeline and mission area. The error prediction model accounts for both satellite elevation dependencies and signal reflection from urban structures in generating the UERE. A primary deliverable of the proposed methodology is the improved ability to predict positioning performance over the intended operational area accounting for all significant ranging error sources. The study confirmed the overall feasibility of the approach and sets the foundation for targeted experimental verification in future work. Another area of future work is the investigation of an optimization strategy to balance navigation performance and trajectory length.

## Figures and Tables

**Table 1:** Summary of simulation results.

	Accuracy (m)		Availability (%)		Distance penalty (%)	
	A*	Nav-A*	A*	Nav-A*	A*	Nav-A*
Scenario-1 Single-frequency	8.1	6.8	39.1	51.7	-	5.8
Scenario-2 Dual-frequency	3.2	2.00	100	63.1		
